# Presence of non-symbiotic yeasts in a symbiont-transferring organ of a stag beetle that lacks yeast symbionts found in other stag beetles

**DOI:** 10.1038/s41598-023-30607-x

**Published:** 2023-03-14

**Authors:** Daichi Yamamoto, Wataru Toki

**Affiliations:** Laboratory of Forest Protection, Graduate School of Bioagricultural Sciences, Nagoya University, Nagoya, Japan

**Keywords:** Biodiversity, Microbial ecology, Symbiosis, Coevolution

## Abstract

Dispersal from wood to wood is essential for wood-inhabiting fungi and wood-inhabiting insects play an important role in the dispersal success of such fungi. However, it is poorly understood whether wood-inhabiting insects which change the habitats from wood to non-wood environments can contribute to the fungal dispersal. Larvae of most stag beetles (Coleoptera: Lucanidae) are wood feeders, while adults are sap feeders. Female adults of lulcanids possess specialized organs (mycetangia) for transportation of fungal symbionts and harbor specific yeasts (e.g., *Scheffersomyces* spp.) within. Here, we report that the lucanid *Aegus subnitidus* harbors non-specific yeasts facultatively in mycetangia. We conducted yeast isolation from mycetangia and hindguts of female adults, in a larval gallery in wood-associated materials, and in female-visiting fermented tree sap using culture-dependent methods. Less than half of the females carried a total of 20 yeast species with small amounts using mycetangia and a female harbored up to five species, suggesting the absence of an intimate association with specific yeasts that are found in other lucanids. Yeast species compositions markedly differed between the larval gallery and sap. Most yeasts from the larval galley exhibited xylose-assimilation abilities, while few yeasts from sap did. Mycetangial yeasts comprised a combination from both sources. In hindguts, most yeasts were found in sap (> 70%) with no yeasts in the larval gallery. Sap-associated yeasts in each female mycetangium were also obtained from the female-visiting sap patch, while mycetangial, larval gallery-associated yeasts were absent from the patch, suggesting the survival success of larval gallery-associated yeasts in mycetangia through sap patches. Therefore, wood-inhabiting insects that possess mycetangia can potentially act as vectors of non-symbiotic wood-inhabiting yeasts dispersing from wood to wood via other environments.

## Introduction

Dispersal from wood to wood is essential for wood-inhabiting fungi. As dead trees are patchily distributed in forests and their occurrence is spatiotemporally unpredictable, animals with a high dispersal ability that use the same habitats can increase the likelihood of dispersal success of the fungi, especially those which have limited dispersal ability. Thus, wood-inhabiting insects are one of the major vectors of wood-inhabiting fungi^1^. For example, all developmental stages of ambrosia beetles (Curculionidae: Scolytinae and Platypodinae) live in wood and only adults temporally go outside to disperse from natal wood to new wood for reproduction. Phoretic wood-inhabiting fungi exist on the exoskeleton of dispersing adults^2^. However, wood-inhabiting insects contain diverse insect taxa with varied ecological traits: some inhabit wood through their life cycle like ambrosia beetles, whereas others change their habitats based on developmental stages. It is poorly understood whether wood-inhabiting insects which change habitats from wood to non-wood environments contribute to fungal dispersal.

In insect-fungus symbioses, diverse insect hosts including Coleoptera, Diptera, and Hymenoptera evolved a specialized pocket-like organ termed the mycetangium or mycangium in their bodies for transportation of fungal symbionts^3–10^. Mycetangia can physically sequester fungal symbionts from outside, and most insect hosts harbor specific fungi in their mycetangia. In ambrosia beetle-fungus systems in wood, moreover, mutualistic fungi outcompete non-mutualistic ones within mycetangia in association with reproductive maturation of the beetles, indicating that their mycetangia function as screening devices of symbionts^11^. Thus, mycetangia may enhance the certainty of dispersal success of the symbionts together with their insect hosts.

**Figure 1:**
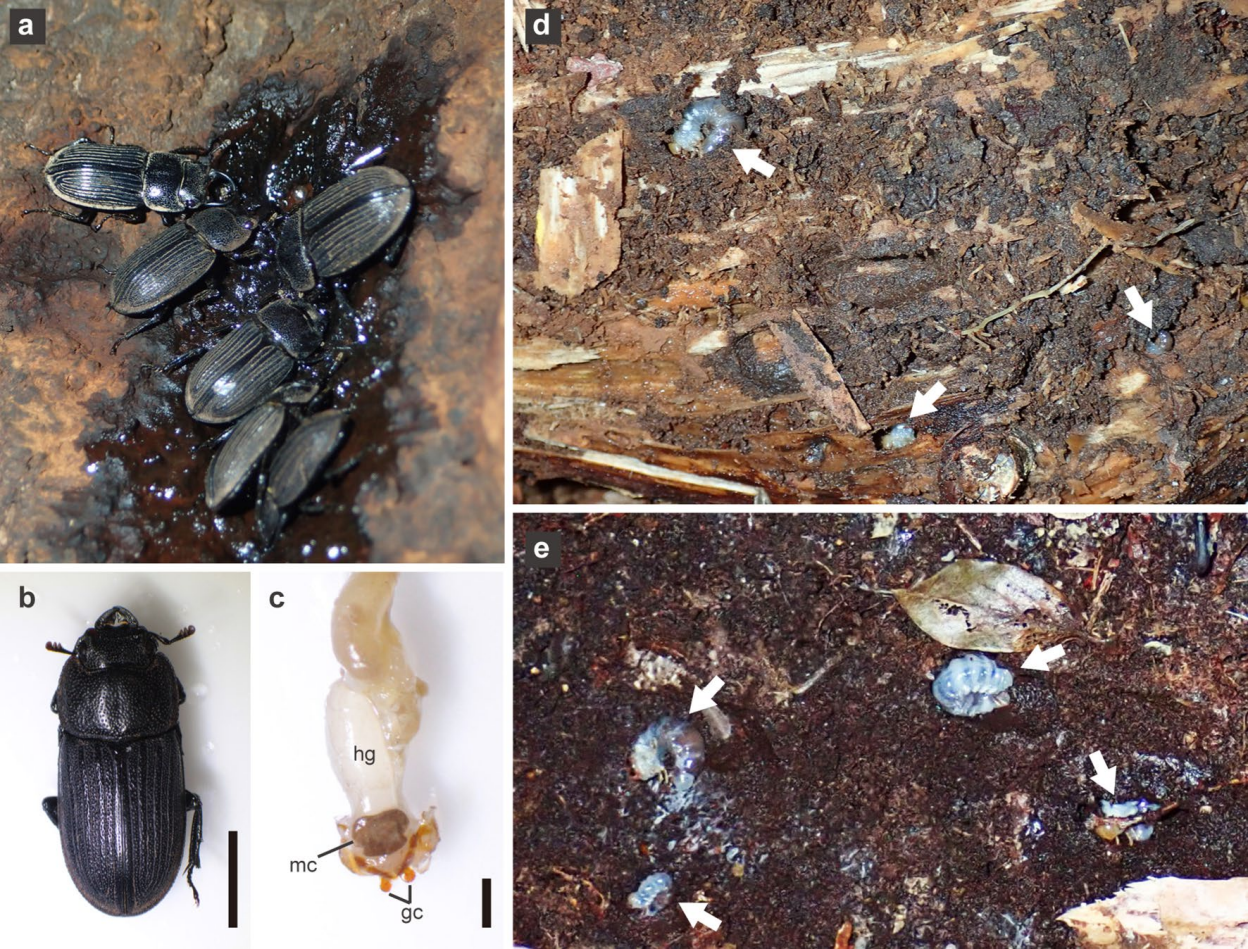
Adults and larvae of *Aegus* *subnitidus.* (**a**) Adults of *A.* *subnitidus* visiting fermented sap of *Quercus* *glauca.* (**b**) An adult female. (**c**) Internal organs dissected from an adult female. (**d**,**e**) Larvae of *A.* *subnitidus* (arrows) in the surface area on the underside of highly decomposed wood of *Pinus* *densiflora* on the ground (**d**) and in the humus in contact with highly decomposed wood of *P.* *densiflora* (**e**). *gc* gonocoxite, *hg* hind gut, *mc* mycetangium. Scale bars = 5.0 mm in (**b**) and 1.0 mm in (**c**).

Adults of most stag beetles (Lucanidae) feed on fermented sap for survival and sexual maturation, whereas larvae are wood feeders (Fig. 1a,d,e). Some lucanids are associated with specific yeasts that can assimilate indigestible sugars including xylose, which is one of the main monosaccharides of hemicelluloses in wood, suggesting that these yeasts aid digestion of wood by larvae^8^. Female adults of lucanids possess mycetangia adjacent to their ovipositors^8^ (Fig. 1c). Their mycetangia are everted immediately after eclosion and are pulled back into the body in pupal chambers; this eversion may be closely related to incorporating yeast symbionts into mycetangia^12^. The mycetangia contain one to three yeast species of the genera *Scheffersomyces*, *Sugiyamaella* and *Yarrowia*^8,13–18^. Given that diverse yeasts are present in fermented sap of trees^19^, their mycetangia, unlike guts, may sequester wood-associated yeast symbionts to avoid contamination by non-symbiotic yeasts, thus enhancing the success of the vertical transmission of symbionts^8^.

Adults of the lucanid *Aegus subnitidus* Waterhouse (formerly, *A. laevicollis subnitidus*) feed on fermented tree sap^20,21^ (Fig. 1a,b). Larvae of the genus *Aegus* inhabit nitrogen-rich wood-associated materials such as termite-infested rotten wood, highly decomposed brown-rotten wood, and humus derived from these woods^22,23^ (Fig. 1d,e). On the other hand, larvae of most lucanids associated with yeasts feed on nitrogen-poor white-rotten wood^22,23^.

Does *A. subnitidus* form symbiotic relationships with specific yeasts like other lucanids? Since the habitat and nutritional food condition of *A. subnitidus* larvae are different from those of white-rotten wood feeders, the association with yeasts such as dependency and specificity may also be different. If dependency on specific yeasts is low in *A. subnitidus*, it is expected that nonspecific yeasts would be recovered from its mycetangia.

Given that individuals of *A. subnitidus* use multiple habitats (i.e., larvae use wood and adults use fermented sap), the yeast community in the mycetangia may be affected by the degree of sequestration of mycetangia. If the mycetangia are physically well-sequestered from outside, they are inaccessible by sap-inhabiting yeasts. Consequently, yeasts in larval galleries may be exclusively present in mycetangia because the opportunity for yeast-loading into a mycetangium is limited to when it is everted upon eclosion in a pupal chamber. If its sequestration is loose, it may be frequently contaminated by sap-inhabiting yeasts when a female visits fermented sap. Moreover, the frequency of such contamination may increase with increasing age of adults because it can be considered that older adults have visited fermented sap more frequently and for longer than younger adults.

In the present study, to determine the potential importance of *A. subnitidus* as a vector of wood-inhabiting yeasts, first, we conducted yeast isolation from mycetangia and hindguts of field-collected female adults. Second, to estimate the origin of each yeast, we also isolated yeasts from larval galleries and fermented tree sap. Third, to estimate the ecological association of isolated yeasts with wood, their xylose-assimilating abilities were investigated as most yeasts that utilize wood-associated materials have this ability^1^. Finally, the evolution and function of mycetangia in stag beetles are discussed.

## Results

### Yeasts in mycetangia and hindguts of female adults, larval galleries, and female-visiting fermented sap

**Figure 2:**
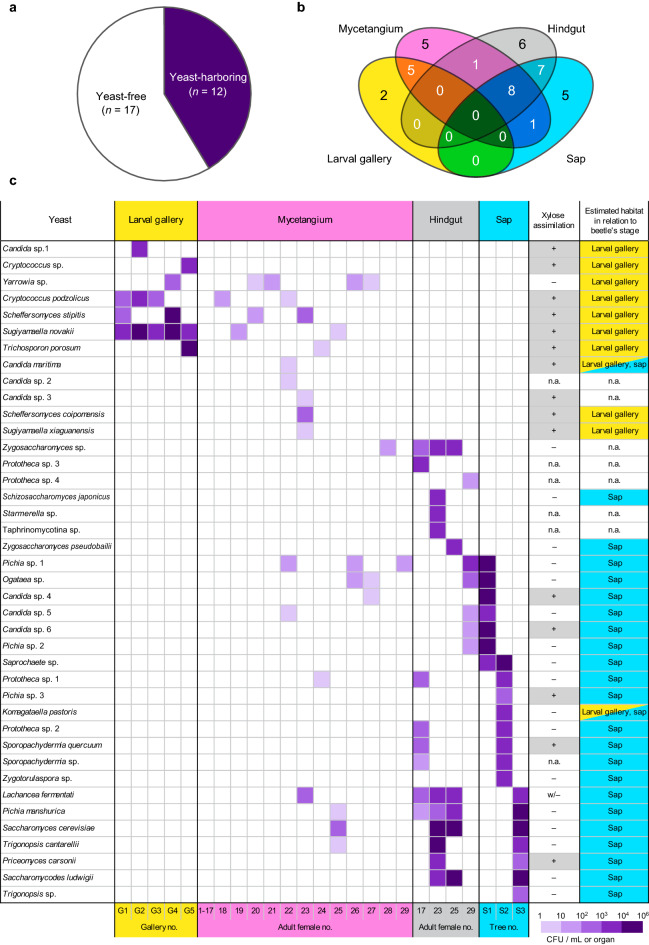
Yeasts isolated from *Aegus* *subnitidus*-associated materials. (**a**) Proportions of yeast-harboring and free females. (**b**) Number of yeast species isolated from each of the isolation sources. **c** Yeasts isolated from larval galleries (G1–G5), mycetangia (1–29), hindguts (17, 23, 25, and 29), and fermented sap of *Quercus* *glauca* (S1), *Q.* *serrata* (S2), and *Q.* *variabilis* (S3). Xylose assimilating ability of each yeast is indicated as follows: +, positive; −, negative; w/−, weakly positive or negative. *n.a.* not applicable.

To determine the yeast community of mycetangia of *A. subnitidus*, we captured 29 female adults (elytral length: mean ± SD = 9.09 ± 0.80 mm, *n* = 29; body weight: 127.4 ± 32.7 mg, *n* = 29) arbitrarily (Table 1). Of those, 12 females harbored yeasts and 17 females did not (Fig. 2a). There was no significant difference in the proportion of yeast-harboring individuals between sexually immature (7/17) and mature (5/12) females (Fisher’s exact test, *P* = 1.00) (Table 1). In total, 20 yeast species excluding unidentified yeasts were isolated from the mycetangia of yeast-harboring females (3.3 to 3.8 × 10^2^ colony forming units (CFU)/mycetangium, *n* = 20) (Supplementary Tables S1, S2, and S5; Fig. 2b,c). The amounts of these yeasts were small compared with other yeast-harboring lucanids^13–18^. Among yeast-harboring females, an individual harbored one to five yeast species. One yeast species was isolated from one to four females (3.4–13.8%).

**Table 1:** *Aegus* *subnitidus* examined in this study.

Yeasts in mycetangia	Number of *Aegus* *subnitidus* obtained from trees^a^					Elytral length (mm)^b^	Body weight (mg)^b^
	*Quercus* *glauca*	*Quercus* *serrata*	*Quercus* *variabilis*	*Ulmus* *davidiana*	Total		
Present	4 (4, 0)	4 (1, 3)	4 (2, 2)	0 (0, 0)	12 (7, 5)	9.09 ± 1.10 (7.22–10.66)	130.1 ± 43.7 (62.7–193.4)
Absent	4 (4, 0)	9 (6, 3)	3 (0, 3)	1 (0, 1)	17 (10, 7)	9.09 ± 0.53 (8.20–10.14)	125.6 ± 23.3 (99.9–176.7)
Total	8 (8, 0)	13 (7, 6)	7 (2, 5)	1 (0, 1)	29 (17, 12)	9.09 ± 0.80 (7.22–10.66)	127.4 ± 32.7 (62.7–193.4)

^a^Numbers of sexually immature and mature individuals are in parentheses, respectively.

^b^Mean ± SD (range).

To determine the yeast community in hindguts of *A. subnitidus*, we examined 4 out of the 29 female adults captured and a total of 22 yeast species were isolated excluding unidentified yeasts (6.7 × 10 to 4.1 × 10^4^ CFU/hindgut, *n* = 22) (Supplementary Tables S1, S2, and S6; Fig. 2b,c). Yeasts were present in hindguts of all four females and a female harbored six to ten yeast species. One yeast species was isolated from one to three females (25.0–75.0%).

From larval galleries of third-instar larvae of *A. subnitidus*, which were constructed inside wood-associated materials, a total of seven yeast species were isolated (5.8 × 10^2^ to 1.4 × 10^4^ CFU/1 mL-gallery-sample-suspension, *n* = 7) (Supplementary Tables S1 and S2; Fig. 2b,c). Yeasts were present in all five galleries and two to three yeast species were detected per gallery. One yeast species was isolated from one to five galleries (20.0–100%).

We also isolated yeasts from female-visiting fermented sap on three oak tree species, *Quercus glauca* (Fagaceae) (S1), *Q. serrata* (S2), and *Q. variabilis* (S3). A total of 21 yeast species excluding unidentified yeasts were isolated (6.7 × 10^2^ to 8.0 × 10^4^ CFU/1 mL-sap-sample-suspension, *n* = 21) (Supplementary Tables S1 and S2; Fig. 2b,c). We detected seven, eight, and seven yeast species from S1, S2, and S3, respectively. In most cases (20/21, 95.2%), each yeast species was isolated from one sap sample only, and the remaining one species was found in two sap samples (S1 and S2).

### Xylose-assimilating ability of isolated yeasts

The ability to assimilate xylose of the isolated yeasts was determined based on the literature or a xylose-assimilation test in culture (Supplementary Tables S3 and S4; Fig. 2c). Among 20 species isolated from mycetangia, 9 showed a positive assimilation ability but 10 did not. The remaining one was not tested due to subsequent unsuccessful culturing. Among 22 species isolated from hindguts, 3 showed a positive assimilation ability but 14 did not. The remaining five were not tested. Six out of seven species obtained from larval galleries showed a positive assimilation ability and the remaining one was negative. Among 21 species obtained from sap, 5 showed a positive assimilation ability and 15 did not. The remaining one was not tested due to subsequent unsuccessful culturing.

### Yeast compositions among mycetangia, hindguts, larval galleries, and sap

Figure 2b shows the number of yeast species isolated from each of the isolation sources. Out of 20 mycetangial yeast species, 5 (25.0%) were isolated from mycetangia only. One (5.0%) was isolated from both mycetangia and hindguts only, which corresponded to 4.5% (1/22) of the yeasts isolated from hindguts. Five (25.0%) were obtained from both mycetangia and larval galleries only, which corresponded to 71.4% (5/7) of the yeasts isolated from larval galleries. One (5.0%) was isolated from both mycetangia and sap only, which corresponded to 4.8% (1/21) of the yeasts isolated from sap. The remaining eight (40.0%) were obtained from three sources: mycetangia, hindguts, and sap, which corresponded to 36.4% (8/22) and 38.1% (8/21) of the yeasts from hindguts and sap, respectively. For hindgut yeasts, six (27.3%) were isolated from hindguts only. Seven (31.2%) were present in hindguts and sap only, which corresponded to 33.3% (7/21) of the yeasts isolated from sap. There were no yeasts isolated from both hindguts and larval galleries or both larval galleries and sap.

When the isolated yeasts were classified as larval gallery-associated or sap-associated according to their isolation records^24–27^ and the results of this study, among 20 mycetangial yeasts, 1 species (5.0%) was both larval gallery- and sap-associated, 7 (35.0%) were larval gallery-associated only, 9 (45.0%) were sap-associated only, and 3 (15.0%) were undetermined (Supplementary Table S3; Fig. 2c). Among 22 yeasts obtained from hindguts, 17 (77.3%) were sap-associated only and 5 (22.7%) were undetermined (Supplementary Table S3; Fig. 2c). All yeasts (100%, 7/7) isolated from larval galleries were larval gallery-associated only, and none of them were isolated from fermented materials. For yeasts obtained from sap, one (4.8%, 1/21) was both larval gallery- and sap-associated, and the others (95.2%, 20/21) were sap-associated only (Supplementary Table S3; Fig. 2c).

Regarding xylose assimilation, 81.8% (9/11) of larval gallery-associated yeasts and 26.1% (6/23) of sap-associated yeasts excluding one species whose ability was undetermined were positive for xylose-assimilating ability (Supplementary Tables S3 and S4; Fig. 2c).

For mycetangial yeasts, among 12 females harboring the yeasts, 4 females carried larval gallery-associated yeast only, 1 female carried sap-associated yeast only, 6 females carried both types of yeasts, and 1 female carried undetermined yeast only (Fig. 2c). Four larval gallery-associated yeasts (*Cryptococcus podzolicus*, *Sc. stipitis*, *Su. novakii*, and *Yarrowia* sp.) were isolated from mycetangia of multiple females derived from multiple tree species (Supplementary Table S5). In the case of sap-associated yeast species isolated from both mycetangia and sap, the insect and sap samples were obtained from the same sap patches (two females—S1, one female—S2, and two females—S3) or different patches on the same tree (two females—near S1) (Supplementary Table S5). For sap-associated yeasts isolated from both the hindgut and sap, the insect and sap samples originated from the same sap patches for three females (one female—S1 and two females—S3) and a female collected at S2 harbored the yeasts isolated from S2 and those from S3 (Supplementary Table S6).

## Discussion

Females of *A. subnitidus* facultatively possessed nonspecific yeasts at a low frequency and with a small amount in their mycetangia irrespective of the degree of sexual maturation. Seven yeast species were found in the larval galleries constructed in highly decayed wood materials and most of them showed xylose-assimilating abilities. More than 70% of larval gallery-associated yeasts were isolated at least once from the mycetangia, but none of them were isolated from the hindguts. In contrast, the yeast community in *A. subnitidus*-visiting tree sap was much more variable and most yeasts did not exhibit xylose-assimilating abilities. Less than half of sap-associated yeasts were recovered from the mycetangia, while more than 70% of them were found in the hindguts. These results suggest that *A. subnitidus* lacks symbiotic associations with specific yeasts found with other stag beetles and that the mycetangia are likely to be physically well-sequestered from the digestive tract and outside but not perfectly, resulting in low-frequency contamination by nonspecific yeasts. Consequently, females of *A. subnitidus* may occasionally carry wood-inhabiting yeasts from wood to wood through fermented sap patches. These data must be interpreted with caution, however, because the yeasts were obtained by culture-dependent methods. The possibility of a symbiotic association between *A. subnitidus* and unculturable yeasts cannot be excluded. In addition, transmission of yeasts from mycetangia to wood was not examined experimentally. Therefore, it is unclear whether mycetangial yeasts of *A. subnitidus* can successfully reach new habitats. Nevertheless, it is notable that 20 species of nonspecific culturable yeasts (including eight larval gallery-associated) were loaded into mycetangia of *A. subnitidus*.

In this study, we roughly estimated the primary habitats of isolated yeasts based on the isolation records^24–27^ and the results of this study. This estimation may be biased or unreliable due to insufficient information on yeast biology in nature. However, yeast species composition and diversity were clearly different between wood (i.e., larval gallery) and sap. Moreover, most larval gallery-derived yeasts had xylose-assimilating abilities, supporting the suggestion that wood-inhabiting yeasts generally assimilate xylose^1^, while most sap-derived yeasts lack this ability. Therefore, this estimation is useful for comparing yeasts among isolation sources, although a detailed inventory of yeasts living in the study site is required to determine the exact primary habitats of the isolated yeasts.

Most larval gallery-associated yeasts were present in larval galleries and mycetangia of *A. subnitidus*, while few of them were isolated from sap and none of them were found in the hindguts (Fig. 2c). Thus, females of *A. subnitidus* possibly incorporate these yeasts into mycetangia within wood-derived materials during eclosion like other lucanids, and then visit fermented sap where sap-associated yeasts are rich and diverse. The larval gallery-associated yeasts may not be released from the mycetangia into sap when the host insects visit sap, or they may be inferior to sap-inhabiting yeasts proliferating in fermented sap. In contrast, sap-associated yeasts constantly dominated the hindguts of female adults. This difference in the yeast community between the mycetangium and hindgut suggests that the hindgut of *A. subnitidus* is not a suitable reservoir for larval gallery-associated yeasts.

Notably, the loading frequency of each yeast species into the mycetangia was low (up to 13.8%) and more than half of female adults examined had ‘empty’ (i.e., culturable yeast-free) mycetangia although several xylose-assimilating yeasts, such as *C. podzolicus*, *Sc. stipitis*, and *Su. novakii*, are likely to be commonly present in the larval galleries (Fig. 2c). In contrast, other lucanids associated with specific, xylose-assimilating yeasts possess the yeast symbionts constantly and abundantly (e.g., *Platycerus* beetle–*Scheffersomyces* yeast)^14,15^. Such low abundance of xylose-assimilating yeasts in mycetangia of *A. subnitidus* suggests asymmetrical relationships between *A. subnitidus* and these yeasts. *Aegus subnitidus* might not depend on the yeasts because the larvae consume nutrient-rich resources, whereas the yeasts might depend on *A. subnitidus* for dispersal, resulting in a phoretic relationship. Alternatively, the larvae may benefit from the yeasts for their growth, and adults can detect yeast-rich wood-associated materials as favorable oviposition substrates without active yeast-loading behavior. Given that post-eclosion behavior of female stag beetles is considered to be closely related to symbiont-loading into mycetangia^12^, behavioral differences in pupal chambers between *A. subnitidus* and other lucanids should be investigated to elucidate what causes the success of yeast loading into mycetangia in the future.

The species compositions of sap-associated yeasts in the mycetangium and hindgut of each female likely reflected the yeast community of the female-visiting sap patch (Fig. 2c). In contrast, the composition of larval gallery-associated yeasts was unlikely to be affected by the patch (Fig. 2c). Although it may be due to the small sample size of sap, these different isolation patterns between ecological types of yeasts suggest that larval gallery-associated yeasts can persist within mycetangia when female adults visit sap patches. It is also suggested that loading of sap-associated yeasts into mycetangia may occur when the adults visit sap patches, but these yeasts may not be maintained for long period inside mycetangia.

Some larval gallery-associated mycetangial yeasts or allied species have been isolated from other stag beetles. *Scheffersomyces coipomensis* and *Yarrowia* sp.-like species were isolated from African *Xiphodontus antilope*^16^, *Su. novakii*-like species from European *Sinodendron cylindricum*, and *Sc. stipitis* from European and Asian *Dorcus* spp.^8,13,14^. These phylogenetically distant yeasts may exhibit ecological or physiological affinity with stag beetles. In the evolutionary time-scale, horizontal transmission of yeast symbionts between stag beetles is suggested^18^. Interestingly, *Sc. stipitis* is vectored by *D. rectus*^8^, which lives sympatrically with *A. subnitidus* in the study site^21^. Although larval microhabitats are different between *D. rectus* and *A. subnitidus*^22,28^, isolation from both lucanid species suggests that *Sc. stipitis* can survive under various conditions of decayed wood using multiple stag beetles as vectors and that horizontal transmission of *Sc. stipitis* between stag beetles may occur frequently.

Previous studies revealed that all examined lucanids including basal lineages possess mycetangia and that their mycetangia contain one to three species of culturable yeasts^8,13–18^. In contrast, a total of 20 species were isolated from mycetangia of *A. subnitidus*. *Aegus* is a phylogenetically derived lineage in Lucanidae^29^, suggesting that mycetangia of *A. subnitidus* retain a fungus-carrying function after loss of mutualism with specific yeasts that are associated with white-rotten wood feeders.

In conclusion, such wood-inhabiting insects that possess mycetangia can potentially act as vectors of non-symbiotic wood-inhabiting fungi from wood to wood via different environments. Further study is required to determine the extent to which such insects contribute to the dispersal of wood-inhabiting fungi and maintenance of fungal diversity in forest ecosystems.

## Materials and methods

### Sampling of insects, larval gallery, and tree sap

All field surveys were conducted in a secondary forest at Higashiyama Campus of Nagoya University, Nagoya, Aichi, Japan (35°09′N, 136°58′E). Adult females of *A. subnitidus* were arbitrarily captured on trunks of *Q. glauca* (8 females from 1 tree), *Q. serrata* (13 females from 4 trees), *Q. variabilis* (7 females from 5 trees), and *Ulmus davidiana* var. *japonica* (Ulmaceae) (1 female from 1 tree) from 17 June to 10 August, 2019. They were weighed and their elytral lengths were determined using digital calipers (Mitutoyo, Kanagawa, Japan). Within 2 days after collection, we dissected all individuals using fire-sterilized tweezers under a stereo-microscope to examine their sexual maturation according to the presence of corpus lutea at the base of ovarioles, which suggests the experience of oviposition^30,31^, and the presence of mature eggs: if either corpus lutea or mature eggs were present, then we judged it as sexually mature; if neither corpus lutea nor mature eggs were present, then we judged it as sexually immature. The dissected insects were subsequently used for yeast isolation.

Larval gallery wall samples were obtained from the gallery surface adjacent to third-instar larvae of *A. subnitidus* (body weight: mean ± SD = 586.3 ± 183.3 mg, *n* = 5) living in the humus in contact with highly decomposed wood of *Pinus densiflora* (Pinaceae) (G1 and G2, *n* = 2) and *Quercus* sp. (G3, *n* = 1), and the surface area on the underside of the same *Quercus* sp. wood (G4, *n* = 1) and other highly decomposed wood of *P. densiflora* (G5, *n* = 1) on 25 January, 2022 (Fig. 1d,e). A piece of the gallery surface (ca. 3 × 3 × 3 mm) was sampled using fire-sterilized tweezers, placed in a sterilized 5-mL tube and stored at 4 °C until use.

Sap samples were obtained from *A. subnitidus* female-present sap patches on the surface of trunks of *Q. glauca* on 29 July, 2019 (S1, *n* = 1), *Q. serrata* on 2 August, 2019 (S2, *n* = 1), and *Q. variabilis* on 10 August, 2019 (S3, *n* = 1). A piece of autoclaved filter paper (10 × 5 mm) was immersed in the sap using fire-sterilized tweezers. Then the paper was placed in a sterilized 2-mL tube and stored at 4 °C until use.

### Yeast isolation

We adopted culture-dependent methods for yeast isolation because all yeasts that have been found from lucanids are culturable on standard culture media for fungi^8,13–18^. The mycetangia of *A. subnitidus* (Fig. 1c) were carefully removed using fire-sterilized tweezers under a stereo-microscope, cut open to expose the inner surface, placed in a 2-mL tube containing 500 or 1000 μL of sterile water, and vortexed vigorously. The hindguts of four adult females (Fig. 1c) were also removed using fire-sterilized tweezers under a stereo-microscope, surface-washed with sterile water for 10 s three times, placed in a 1.5-mL tube containing 500 μL of sterile water, and ground using an autoclaved pestle, and then, 10- and 100-fold diluted suspensions were made. For the larval gallery and sap, within 3 days after collection, 1000 μL of sterile water was added into the gallery- or sap-containing tube, the tubes were vortexed vigorously, and the 10-, 100-, and 1000-fold diluted suspensions were made. Then, 50 or 100 μL of the suspension was spread over potato dextrose agar (PDA) (Difco, Detroit, MI, USA) plates containing 20 μg/mL of rifampicin (Wako, Osaka, Japan) (3 replicates). The plates were incubated at 25 °C in the dark for 2 days. The fungal colonies that grew on the plates were roughly classified based on their morphological traits (morphotype), and CFU of each morphotype were calculated. Up to eight colonies of each morphotype were selected arbitrarily for DNA analysis and individually transferred to new PDA plates. When a morphotype contained two or more species, CFU of each species were recalculated according to the proportion of the species in the selected colonies.

Frequencies of yeast-present mycetangia were compared between sexually immature and mature individuals using Fisher’s exact test. Calculations were performed using R 4.0.5^32^.

### Identification of yeasts

DNA sequences (ca. 600 bps) in the D1/D2 domain of the 26S rRNA gene (26S) were determined to identify yeast isolates obtained from mycetangia, hindguts, larval galleries, and tree sap. For representative isolates, additionally, we determined the DNA sequences of the internal transcribed spacer region and 5.8S rRNA gene (ITS/5.8S) (ca. 600 bps) and those of the translation elongation factor-1α gene (TEF) (ca. 800 bps).

For DNA extraction, small pellets of fungal colonies were suspended in 100 μL of TE buffer and incubated at 99 °C for 10 min. After centrifugation, 30 μL of the supernatant containing DNA was stored and directly used for PCR amplification. The following primer pairs were used for PCR: LS1 (5′-AGTACCCGCTGAACTTAAG-3′) (forward)^33^ and NL4 (5′-GGTCCGTGTTTCAAGACGG-3′) (reverse)^34^ for 26S, ITS5 (5′-GGAAGTAAAAGTCGTAACAAGG-3′) (forward) and ITS4 (5′-TCCTCCGCTTATTGATATGC-3′) (reverse)^35^ for ITS/5.8S, and YTEF-1G (5′-GGTAAGGGTTCTTTCAAGTACGCTTGGG-3′) (forward) and YTEF-6G (5′-CGTTCTTGGAGTCACCACAGACGTTACCTC-3′) (reverse)^36^ for TEF.

The PCR products were purified using Exo SAP-IT (Thermo Fisher Scientific, Waltham, MA, USA), and directly sequenced using BigDye Terminators (Thermo Fisher Scientific) and ABI PRISM 3130xl Genetic Analyzer (Thermo Fisher Scientific). Nucleotide sequence data reported in this study have been deposited in the DNA Data Bank of Japan (DDBJ)/EMBL/GenBank with accession numbers LC661389–LC661454, LC661588–LC661618, LC704689–LC704693, LC744337–LC744358 (Supplementary Table S1). The sequences were subjected to BLASTn searches^37^ for identification.

### Carbon assimilation test

For 20 yeast species whose xylose-assimilating abilities were unknown, representative isolates were cultured aerobically in 20 mL of yeast nitrogen base (YNB) (Difco) containing 0.5% glucose at 25 °C in the dark for 3 days. The culture media were centrifuged and cell pellets were suspended in sterile water, in which OD_600_ was adjusted to 0.04–0.16. Then, 50 μL of the cell suspension was added into a tube (2 mL) with 1 mL of each of three different media containing YNB and one of the following carbon sources: d-glucose, d-xylose, and no carbon source (*n* = 3–4). The concentration of each carbon source was 0.5 g/L. The tubes were shaken at 85 rpm and incubated at 25 °C in the dark for 7 days. Afterwards the presence of visible pellets of yeasts and OD_600_ were recorded to determine the growth of each strain. The degree of assimilation was scored according to the presence of the pellets and difference of the turbidity increase (ΔOD_600_) between culture media containing no and a given carbon source^38^.

### Ecological association of yeasts with wood and sap

Primary habitats of isolated yeasts were estimated according to their isolation records including those in the present study^24–27^. A yeast was classified as a larval gallery-associated yeast when it had been identified from the wood, wood-inhabiting insects or larval gallery of *A. subnitidus*. When a yeast was isolated from fermented materials or *A. subnitidus*-visiting tree sap, it was designated as a sap-associated yeast.

## Supplementary Information

Supplementary Tables.

## Data Availability

The datasets presented in this study can be found in DDBJ/EMBL/GenBank (accession numbers: LC661389–LC661454, LC661588–LC661618, LC704689–LC704693, LC744337–LC744358).
