# Supplementary material for: Presence of non-symbiotic yeasts in a symbiont-transferring organ of a stag beetle that lacks yeast symbionts found in other stag beetles
Source: Sci Rep. 2023 Mar 14;13:3726. doi: 10.1038/s41598-023-30607-x (PMC10014939; doi:10.1038/s41598-023-30607-x)
Supplement: Supplementary file 1 — Supplementary Tables. [file 41598_2023_30607_MOESM1_ESM.pdf]

Supplementary information

Presence of non-symbiotic yeasts in a symbiont-transferring organ of a stag beetle that lacks yeast symbionts found in other stag beetles

Daichi Yamamoto and Wataru Toki

Supplementary Table S1. DDBJ/EMBL/GenBank accession numbers of each *Aegus subnitidus*-related yeast strain sequenced.

Strain	Estimated taxon	Accession no.			Maximum partial similarity (%)		
		26S	ITS/5.8S	TEF	26S	ITS/5.8S	TEF
Af110-2-1	*Candida maritima*	LC661390	LC661425	LC661589	99.2	98.2	97.5
AS2-5-1	*Candida* sp. 1	LC704690	LC704693	n.a.	99.8	99.0	n.a.
Af110-3-1	*Candida* sp. 2	LC661391	LC661426	LC661590	98.5	95.1	92.2
Af146-4-1	*Candida* sp. 3	LC661409	LC661442	LC661604	99.7	89.9	89.1
Af116-2-1	*Candida* sp. 4	LC661397	LC661431	LC661593	95.9	82.8	93.1
Af110-3-2	*Candida* sp. 5	LC661392	LC661427	LC661591	98.4	84.0	91.7
Af135S-6-1	*Candida* sp. 6	LC661411	LC661444	LC661606	99.8	87.7	86.8
Af114-1-1	*Cryptococcus podzolicus*	LC661395	LC661429	n.a.	99.7	100	n.a.
AS2-4-2	*Cryptococcus podzolicus*	LC704689	LC704692	n.a.	100	99.8	n.a.
AS5-4-1	*Cryptococcus* sp.	LC704691	n.a.	n.a.	100	n.a.	n.a.
Af140S-6-4	*Komagataella pastoris*	LC661418	LC661449	LC661612	99.7	100	98.8
Af146-1-4	*Lachancea fermentati*	LC661407	LC661440	LC661603	100	100	98.7
Af116-2-2	*Ogataea* sp.	LC661398	LC661432	LC661594	97.3	89.7	89.3
Af145-3-1	*Pichia manshurica*	LC661404	n.a.	LC661600	100	n.a.	98.8
Af146S-3-1	*Pichia manshurica*	n.a.	LC661452	LC661616	n.a.	99.6	98.8
Af135-1-1	*Pichia* sp. 1	LC661400	LC661434	LC661596	99.7	88.8	92.4
Af135S-3-1	*Pichia* sp. 2	LC661410	LC661443	LC661605	100	98.8	97.7
Af140S-3-1	*Pichia* sp. 3	LC661415	LC661447	LC661610	100	99.2	99.1
Af146S-5-2	*Priceomyces carsonii*	LC661423	LC661454	LC661618	100	99.8	91.1
Af113-2-1	*Prototheca* sp. 1	LC661394	n.a.	n.a.	98.5	n.a.	n.a.
Af140Hg-113x-5	*Prototheca* sp. 1	LC744340	n.a.	n.a.	98.7	n.a.	n.a.
Af140S-5-2	*Prototheca* sp. 2	LC661417	n.a.	n.a.	100	n.a.	n.a.
Af140Hg-113x-4	*Prototheca* sp. 2	LC744339	n.a.	n.a.	99.5	n.a.	n.a.
Af140Hg-113x-6	*Prototheca* sp. 3	LC744341	n.a.	n.a.	98.0	n.a.	n.a.
Af135Hg-4-1	*Prototheca* sp. 4	LC744337	n.a.	n.a.	100	n.a.	n.a.
Af145-1-1	*Saccharomyces cerevisiae*	LC661402	LC661436	LC661598	100	100	100
Af146S-2-1	*Saccharomycodes ludwigii*	LC661420	n.a.	LC661614	99.8	n.a.	97.5
Af146S-2-2	*Saccharomycodes ludwigii*	LC661421	LC661451	LC661615	100	99.4	97.3
Af146S-1x-1	*Saccharomycodes ludwigii*	LC661419	LC661450	LC661613	99.5	99.2	99.1
Af145Hg-2-1	*Saccharomycodes ludwigii*	LC744343	n.a.	n.a.	99.8	n.a.	n.a.
Af145Hg-2-2	*Saccharomycodes ludwigii*	LC744344	LC744353	n.a.	99.7	99.2	n.a.

Af145Hg-2-3	*Saccharomycodes ludwigii*	LC744345	n.a.	n.a.	99.7	n.a.	n.a.
Af145Hg-2-5	*Saccharomycodes ludwigii*	LC744346	LC744354	n.a.	99.7	99.1	n.a.
Af140S-1-3	*Saprochaete* sp.	LC661413	LC661445	LC661608	100	92.0	94.7
Af146-1-1	*Scheffersomyces coipomensis*	LC661406	LC661439	LC661602	100	100	97.9
Af118-1-4	*Scheffersomyces stipitis*	LC661399	LC661433	LC661595	100	100	98.9
Af146Hg-6-1	*Schizosaccharomyces japonicus*	LC744349	LC744357	n.a.	100	100	n.a.
Af140S-4-1	*Sporopachydermia quercuum*	LC661416	LC661448	LC661611	100	99.8	88.8
Af140S-113x-1	*Sporopachydermia* sp.	LC661412	n.a.	LC661607	98.9	n.a.	88.0
Af140Hg-1-8	*Sporopachydermia* sp.	LC744338	LC744351	n.a.	99.1	98.8	n.a.
Af146Hg-B-1	*Starmerella* sp.	LC744350	LC744358	n.a.	94.9	89.7	n.a.
Af141-1-1	*Sugiyamaella novakii*	LC661401	LC661435	LC661597	100	100	90.7
Af145-2-1	*Sugiyamaella novakii*	LC661403	LC661437	LC661599	100	99.8	90.6
Af146-3-1	*Sugiyamaella xiaguanensis*	LC661408	LC661441	n.a.	100	99.6	n.a.
Af146Hg-4-7	Taphrinomycotina sp.	LC744348	LC744356	n.a.	88.6	95.4	n.a.
Af113-1-1	*Trichosporon porosum*	LC661393	LC661428	n.a.	100	100	n.a.
Af145-4-1	*Trigonopsis cantarellii*	LC661405	LC661438	LC661601	100	100	88.3
Af146S-5-1	*Trigonopsis* sp.	LC661422	LC661453	LC661617	98.2	96.4	88.6
Af116-1-1	*Yarrowia* sp.	LC661396	LC661430	LC661592	98.5	89.7	90.4
Af145Hg-4-1	*Zygosaccharomyces pseudobailii*	LC744347	LC744355	n.a.	100	100	n.a.
Af106-1-1	*Zygosaccharomyces* sp.	LC661389	LC661424	LC661588	100	99.8	93.8
Af145Hg-1-3	*Zygosaccharomyces* sp.	LC744342	LC744352	n.a.	99.8	99.8	n.a.
Af140S-2-1	*Zygotorulaspora* sp.	LC661414	LC661446	LC661609	100	99.7	93.9

n.a., not applicable.

Supplementary Table S2. Yeasts isolated from larval galleries, mycetangia, and hindguts of *Aegus subnitidus* and *A. subnitidus* visiting fermented sap of oak trees.

Yeast strain	Isolation source												
	Larval galleries of *Aegus subnitidus*			Mycetangia of *Aegus subnitidus*			Hindguts of *Aegus subnitidus*			Fermented tree sap			
		CFU/mL			CFU/mycetangium			CFU/hindgut			CFU/mL		
	Frequency (%)	Range[a]	Mean ± SD[a]	Frequency (%)	Range[a]	Mean ± SD[a]	Frequency (%)	Range[a]	Mean ± SD[a]	Frequency (%)	*Quercus glauca* (S1)	*Quercus serrata* (S2)	*Quercus variabilis* (S3)
Candida sp. 1 (AS2-5-1)	1/5 (20.0)	2.7×10^3	2.7×10^3	0/29 (0)	0	0	0/4 (0)	0	0	0/3 (0)	0	0	0
Cryptococcus sp. (AS5-4-1)	1/5 (20.0)	1.4×10^3	1.4×10^3	0/29 (0)	0	0	0/4 (0)	0	0	0/3 (0)	0	0	0
Yarrowia sp. (Af116-1-1)	1/5 (20.0)	6.7×10^2	6.7×10^2	4/29 (13.8)	$3.3 - 4.0 \times 10$	$1.7 \times 10 \pm 1.7 \times 10$	0/4 (0)	0	0	0/3 (0)	0	0	0
Cryptococcus podzolicus (Af114-1-1, AS2-4-2)	3/5 (60.0)	$5.8 \times 10^2 - 4.0 \times 10^3$	$1.8 \times 10^3 \pm 1.9 \times 10^3$	2/29 (6.9)	$6.7 - 1.0 \times 10$	8.3 ± 2.4	0/4 (0)	0	0	0/3 (0)	0	0	0
Scheffersomyces stipitis (Af118-1-4)	2/5 (40.0)	$6.7 \times 10^3 - 1.4 \times 10^4$	$7.3 \times 10^3 \pm 9.4 \times 10^3$	2/29 (6.9)	$2.3 \times 10 - 1.6 \times 10^2$	$9.3 \times 10 \pm 9.9 \times 10$	0/4 (0)	0	0	0/3 (0)	0	0	0
Sugiyamaella novakii (Af141-1-1, Af145-2-1)	5/5 (100)	$1.9 \times 10^3 - 1.5 \times 10^5$	$3.6 \times 10^4 \pm 6.6 \times 10^4$	2/29 (6.9)	$3.3 - 1.0 \times 10$	6.7 ± 4.7	0/4 (0)	0	0	0/3 (0)	0	0	0
Trichosporon porosum (Af113-1-1)	1/5 (20.0)	1.1×10^4	1.1×10^4	1/29 (3.4)	3.3	3.3	0/4 (0)	0	0	0/3 (0)	0	0	0
Candida maritima (Af110-2-1)	0/5 (0)	0	0	1/29 (3.4)	3.3	3.3	0/4 (0)	0	0	0/3 (0)	0	0	0

Candida sp. 2 (Af110-3-1)	0/5 (0)	0	0	1/29 (3.4)	3.3	3.3	0/4 (0)	0	0	0/3 (0)	0	0	0
Candida sp. 3 (Af146-4-1)	0/5 (0)	0	0	1/29 (3.4)	3.3	3.3	0/4 (0)	0	0	0/3 (0)	0	0	0
Scheffersomyces coipomensis (Af146-1-1)	0/5 (0)	0	0	1/29 (3.4)	3.8×10^2	3.8×10^2	0/4 (0)	0	0	0/3 (0)	0	0	0
Sugiyamaella xiaguanensis (Af146-3-1)	0/5 (0)	0	0	1/29 (3.4)	6.7	6.7	0/4 (0)	0	0	0/3 (0)	0	0	0
Zygosaccharomyces sp. (Af106-1-1, Af145Hg-1-3)	0/5 (0)	0	0	1/29 (3.4)	1.7×10	1.7×10	3/4 (75.0)	1.3×10^2 – 6.8×10^3	$3.3 \times 10^3 \pm 3.4 \times 10^3$	0/3 (0)	0	0	0
Prototheca sp. 3 (Af140Hg-113x-6)	0/5 (0)	0	0	0/29 (0)	0	0	1/4 (25.0)	1.9×10^3	1.9×10^3	0/3 (0)	0	0	0
Prototheca sp. 4 (Af135Hg-4-1)	0/5 (0)	0	0	0/29 (0)	0	0	1/4 (25.0)	6.7×10	6.7×10	0/3 (0)	0	0	0
Schizosaccharomyces japonicus (Af146Hg-6-1)	0/5 (0)	0	0	0/29 (0)	0	0	1/4 (25.0)	4.0×10^3	4.0×10^3	0/3 (0)	0	0	0
Starmerella sp. (Af146Hg-B-1)	0/5 (0)	0	0	0/29 (0)	0	0	1/4 (25.0)	1.3×10^3	1.3×10^3	0/3 (0)	0	0	0
Taphrinomycotina sp. (Af146Hg-4-7)	0/5 (0)	0	0	0/29 (0)	0	0	1/4 (25.0)	1.5×10^3	1.5×10^3	0/3 (0)	0	0	0
Zygosaccharomyces pseudobailii (Af145Hg-4-1)	0/5 (0)	0	0	0/29 (0)	0	0	1/4 (25.0)	2.0×10^3	2.0×10^3	0/3 (0)	0	0	0
Pichia sp. 1 (Af135-1-1)	0/5 (0)	0	0	3/29 (10.3)	1.7×10 – 3.7×10	$2.4 \times 10 \pm 1.1 \times 10$	1/4 (25.0)	9.3×10^3	9.3×10^3	1/3 (33.3)	3.3×10^4	0	0

Ogataea sp. (Af116-2-2)	0/5 (0)	0	0	2/29 (6.9)	3.3 − 1.7 × 10	1.0 × 10 ± 9.4	1/4 (25.0)	1.3×10^2	1.3×10^2	1/3 (33.3)	8.0×10^4	0	0
Candida sp. 4 (Af116-2-1)	0/5 (0)	0	0	1/29 (3.4)	3.3	3.3	0/4 (0)	0	0	1/3 (33.3)	1.3×10^4	0	0
Candida sp. 5 (Af110-3-2)	0/5 (0)	0	0	1/29 (3.4)	3.3	3.3	1/4 (25.0)	6.7 × 10	6.7 × 10	1/3 (33.3)	6.7×10^3	0	0
Candida sp. 6 (Af135S-6-1)	0/5 (0)	0	0	0/29 (0)	0	0	1/4 (25.0)	6.7 × 10	6.7 × 10	1/3 (33.3)	2.7×10^4	0	0
Pichia sp. 2 (Af135S-3-1)	0/5 (0)	0	0	0/29 (0)	0	0	1/4 (25.0)	6.7 × 10	6.7 × 10	1/3 (33.3)	3.3×10^4	0	0
Saprochaete sp. (Af140S-1-3)	0/5 (0)	0	0	0/29 (0)	0	0	0/4 (0)	0	0	2/3 (66.7)	6.7×10^3	1.9×10^4	0
Prototheca sp. 1 (Af113-2-1, Af140Hg-113x-5)	0/5 (0)	0	0	1/29 (3.4)	3.3	3.3	1/4 (25.0)	3.8×10^2	3.8×10^2	1/3 (33.3)	0	1.0×10^3	0
Pichia sp. 3 (Af140S-3-1)	0/5 (0)	0	0	0/29 (0)	0	0	0/4 (0)	0	0	1/3 (33.3)	0	6.7×10^2	0
Komagataella pastoris (Af140S-6-4)	0/5 (0)	0	0	0/29 (0)	0	0	0/4 (0)	0	0	1/3 (33.3)	0	4.7×10^3	0
Prototheca sp. 2 (Af140S-5-2, Af140Hg-113x-4)	0/5 (0)	0	0	0/29 (0)	0	0	1/4 (25.0)	7.5×10^2	7.5×10^2	1/3 (33.3)	0	1.3×10^3	0
Sporopachydermia quercuum (Af140S-4-1)	0/5 (0)	0	0	0/29 (0)	0	0	1/4 (25.0)	1.3×10^2	1.3×10^2	1/3 (33.3)	0	2.0×10^3	0

Sporopachydermia sp. (Af140S-113x-1, Af140Hg-1-8)	0/5 (0)	0	0	0/29 (0)	0	0	1/4 (25.0)	6.7 × 10	6.7 × 10	1/3 (33.3)	0	1.0×10^3	0
Zygotorulaspora sp. (Af140S-2-1)	0/5 (0)	0	0	0/29 (0)	0	0	0/4 (0)	0	0	1/3 (33.3)	0	2.0×10^3	0
Lachancea fermentati (Af146-1-4)	0/5 (0)	0	0	1/29 (3.4)	1.3×10^2	1.3×10^2	3/4 (75.0)	2.0×10^2 – 6.8×10^3	$4.2 \times 10^3 \pm 3.5 \times 10^3$	1/3 (33.3)	0	0	4.8×10^3
Pichia manshurica (Af145-3-1, Af146S-3-1)	0/5 (0)	0	0	1/29 (3.4)	3.3	3.3	3/4 (75.0)	6.7 × 10 – 1.3×10^3	$6.9 \times 10^2 \pm 6.3 \times 10^2$	1/3 (33.3)	0	0	1.0×10^4
Saccharomyces cerevisiae (Af145-1-1)	0/5 (0)	0	0	1/29 (3.4)	1.7×10^2	1.7×10^2	2/4 (50.0)	1.4×10^4 – 4.1×10^4	$2.8 \times 10^4 \pm 1.9 \times 10^4$	1/3 (33.3)	0	0	1.5×10^4
Trigonopsis cantarellii (Af145-4-1)	0/5 (0)	0	0	1/29 (3.4)	3.3	3.3	1/4 (25.0)	1.1×10^4	1.1×10^4	1/3 (33.3)	0	0	3.3×10^3
Priceomyces carsonii (Af146S-5-2)	0/5 (0)	0	0	0/29 (0)	0	0	1/4 (25.0)	1.3×10^3	1.3×10^3	1/3 (33.3)	0	0	6.7×10^2
Saccharomycodes ludwigii (Af146S-2-1, 2, Af146S-1x-1, Af145Hg-2-1, 2, 3, 5)	0/5 (0)	0	0	0/29 (0)	0	0	2/4 (50.0)	2.0×10^3 – 2.8×10^4	$1.5 \times 10^4 \pm 1.8 \times 10^4$	1/3 (33.3)	0	0	1.1×10^4
Trigonopsis sp. (Af146S-5-1)	0/5 (0)	0	0	0/29 (0)	0	0	0/4 (0)	0	0	1/3 (33.3)	0	0	6.7×10^2

[a] Values are based on yeast-present samples.

Supplementary Table S3. Isolation records and xylose-assimilating abilities of yeasts isolated from *Aegus subnitidus*-related materials.

Yeast strain	Isolation record[a]	Xylose assimilation[b]	Reference[c]
Candida sp. 1 (AS2-5-1)	n.a.	+	5
Cryptococcus sp. (AS5-4-1)	n.a.	+	5
Yarrowia sp. (Af116-1-1)	n.a.	–	5
Cryptococcus podzolicus (Af114-1-1, AS2-4-2)	S	+	2
Scheffersomyces stipitis (Af118-1-4)	W	+	2
Sugiyamaella novakii (Af141-1-1, Af145-2-1)	W	+	2
Trichosporon porosum (Af113-1-1)	S, W	+	2
Candida maritima (Af110-2-1)	F, W	+	1, 2
Candida sp. 2 (Af110-3-1)	n.a.	n.a.	n.a.
Candida sp. 3 (Af146-4-1)	n.a.	+	5
Scheffersomyces coipomensis (Af146-1-1)	W	+	2
Sugiyamaella xiaguanensis (Af146-3-1)	W	+	4
Zygosaccharomyces sp. (Af106-1-1, Af145Hg-1-3)	n.a.	–	5
Prototheca sp. 3 (Af140Hg-113x-6)	n.a.	n.a.	n.a.
Prototheca sp. 4 (Af135Hg-4-1)	n.a.	n.a.	n.a.
Schizosaccharomyces japonicus (Af146Hg-6-1)	F	–	2
Starmerella sp. (Af146Hg-B-1)	n.a.	n.a.	n.a.
Taphrinomycotina sp. (Af146Hg-4-7)	n.a.	n.a.	n.a.
Zygosaccharomyces pseudobailii (Af145Hg-4-1)	F	–	3
Pichia sp. 1 (Af135-1-1)	n.a.	–	5
Ogataea sp. (Af116-2-2)	n.a.	–	5
Candida sp. 4 (Af116-2-1)	n.a.	+	5
Candida sp. 5 (Af110-3-2)	n.a.	–	5
Candida sp. 6 (Af135S-6-1)	n.a.	+	5
Pichia sp. 2 (Af135S-3-1)	n.a.	–	5
Saprochaete sp. (Af140S-1-3)	n.a.	–	5
Prototheca sp. 1 (Af113-2-1, Af140Hg-113x-5)	n.a.	–	5
Pichia sp. 3 (Af140S-3-1)	n.a.	+	5
Komagataella pastoris (Af140S-6-4)	F, W	–	1, 2, 5
Prototheca sp. 2 (Af140S-5-2, Af140Hg-113x-4)	n.a.	–	5
Sporopachydermia quercuum (Af140S-4-1)	F	+	2
Sporopachydermia sp. (Af140S-113x-1, Af140Hg-1-8)	n.a.	n.a.	n.a.

Zygotorulaspora sp. (Af140S-2-1)	n.a.	–	2, 5
Lachancea fermentati (Af146-1-4)	F	w/–	2, 5
Pichia manshurica (Af145-3-1, Af146S-3-1)	F	–	2
Saccharomyces cerevisiae (Af145-1-1)	F	–	1, 2
Trigonopsis cantarellii (Af145-4-1)	F	–	2, 5
Priceomyces carsonii (Af146S-5-2)	F, S[d]	+	2
Saccharomycodes ludwigii (Af146S-2-1, 2, Af146S-1x-1, Af145Hg-2-1, 2, 3, 5)	F, S[d]	–	2
Trigonopsis sp. (Af146S-5-1)	n.a.	–	5

n.a., not applicable.

[a] F, fermented materials; S, soil; W, wood and/or wood-inhabiting insects.

[b] –, negative; w/– weakly positive or negative; +, positive.

[c] 1, [24]; 2, [25]; 3, [26]; 4, [27]; 5, this study.

[d] Soil in a garden or orchard where *A. subnitidus* is unlikely to live.

Supplementary Table S4. Growth of yeasts isolated from *Aegus subnitidus*-related materials on glucose and xylose.

Yeast strain	Glucose		Xylose	
	ΔOD_{600}[a]	Growth[b]	ΔOD_{600}[a]	Growth[b]
Candida sp. 1 (AS2-5-1)	**0.54**	++	**0.55**	++
Candida sp. 3 (Af146-4-1)	**0.38**	+	**0.34**	+
Candida sp. 4 (Af116-2-1)	**0.48**	++	**0.68**	++
Candida sp. 5 (Af110-3-2)	**0.79**	++	–0.03	–
Candida sp. 6 (Af135S-6-1)	**0.75**	++	**0.73**	++
Cryptococcus sp. (AS5-4-1)	**0.57**	++	**0.55**	++
Komagataella pastoris (Af140S-6-4)	**0.67**	++	0.02	–
Lachancea fermentati (Af146-1-4)	**0.69**	++	0.08	w/–
Ogataea sp. (Af116-2-2)	**0.67**	++	0.04	–
Pichia sp. 1 (Af135-1-1)	**0.56**	++	–0.01	–
Pichia sp. 2 (Af135S-3-1)	**0.67**	++	–0.02	–
Pichia sp. 3 (Af140S-3-1)	**0.67**	++	**0.25**	+
Prototheca sp. 1 (Af113-2-1)	**0.53**	++	**0.01**	–
Prototheca sp. 2 (Af140S-5-2)	**0.18**	+	0	–
Saprochaete sp. (Af140S-1-3)	**0.23**	+	0	–
Trigonopsis cantarellii (Af145-4-1)	**0.08**	w	**0.01**	–
Trigonopsis sp. (Af146S-5-1)	**0.70**	++	0.04	–
Yarrowia sp. (Af116-1-1)	**0.29**	+	–0.01	–
Zygosaccharomyces sp. (Af106-1-1)	**0.48**	++	**0.01**	–
Zygotorulaspora sp. (Af140S-2-1)	**0.21**	+	**0.01**	–

[a] Difference in the turbidity increase between culture media containing no and a given carbon source. Data in bold indicate that pellets of yeasts could be observed.
[b] –, no growth; w, weak growth; w/–, weak or no growth; +, moderate growth; ++, strong growth.

Supplementary Table S5. Mycetangial yeasts of *Aegus subnitidus* obtained from fermented sap patches of trees.

Individual ID (no. in Fig. 2c)	Elytral length (mm)	Body weight (mg)	Sexual maturation	Sampling date	Tree			Yeast in mycetangia	
					Species	Individual	Sap ID[a]	Number of species	Species (other isolation sources in environments)[b]
Af110 (22)	9.17	148.9	Immature	25 June, 2019	*Quercus glauca*	GB01	S1	5	*Candida maritima*
									Candida sp. 2
									Candida sp. 5 (S1)
									Cryptococcus podzolicus (G1-G3)
									Pichia sp. 1 (S1)
Af135 (29)	10.13	193.4	Immature	29 July, 2019	*Q. glauca*	GB01	S1	1	*Pichia* sp. 1 (S1)
Af103 (2)	8.55	125.6	Immature	17 June, 2019	*Q. glauca*	GB01	Non-recorded patch located near S1	0	n.a.
Af101 (1)	8.20	99.9	Immature	17 June, 2019	*Q. glauca*	GB01	A324	0	n.a.
Af102 (26)	7.46	79.7	Immature	17 June, 2019	*Q. glauca*	GB01	A323	3	*Ogataea* sp. (S1)
									Pichia sp. 1 (S1)
									Yarrowia sp. (G4)
Af108 (6)	8.71	105.1	Immature	21 June, 2019	*Q. glauca*	GB01	A323	0	n.a.
Af109 (7)	9.02	101.9	Immature	25 June, 2019	*Q. glauca*	GB01	A323	0	n.a.
Af116 (27)	8.50	110.9	Immature	2 July, 2019	*Q. glauca*	GB01	A323	3	*Candida* sp. 4 (S1)
									Ogataea sp. (S1)
									Yarrowia sp. (G4)
Af104 (3)	8.50	108.3	Immature	17 June, 2019	*Q. serrata*	SE01	S2	0	n.a.

Af111 (8)	9.04	134.2	Immature	25 June, 2019	*Q. serrata*	SE01	S2	0	n.a.
Af112 (9)	8.44	101.4	Immature	25 June, 2019	*Q. serrata*	SE01	S2	0	n.a.
Af113 (24)	8.52	94.1	Immature	25 June, 2019	*Q. serrata*	SE01	S2	2	*Prototheca* sp. 1 (S2)
									Trichosporon porosum (G5)
Af128 (14)	9.03	117.1	Mature	17 July, 2019	*Q. serrata*	SE01	S2	0	n.a.
Af129 (15)	9.70	171.2	Mature	17 July, 2019	*Q. serrata*	SE01	S2	0	n.a.
Af140 (17)	9.53	126.5	Immature	2 August, 2019	*Q. serrata*	SE01	S2	0	n.a.
Af141 (19)	10.66	173.8	Mature	2 August, 2019	*Q. serrata*	SE01	S2	1	*Sugiyamaella novakii* (G1-G5)
Af105 (4)	8.94	105.5	Immature	18 June, 2019	*Q. serrata*	SB01	Not on a sap patch	0	n.a.
Af125 (21)	7.22	62.7	Mature	17 July, 2019	*Q. serrata*	SB01	A284	1	*Yarrowia* sp. (G4)
Af115 (10)	9.69	151.6	Mature	2 July, 2019	*Q. serrata*	ST01	A288	0	n.a.
Af136 (16)	9.60	125.8	Immature	29 July, 2019	*Q. serrata*	ST01	A288	0	n.a.
Af118 (20)	9.22	125.2	Mature	10 July, 2019	*Q. serrata*	Not recorded	Non-recorded sap patch	2	*Scheffersomyces stipitis* (G1, G4)
									Yarrowia sp. (G4)
Af126 (13)	10.14	176.7	Mature	17 July, 2019	*Q. variabilis*	VD01	S3	0	n.a.
Af145 (25)	10.46	187.0	Mature	10 August, 2019	*Q. variabilis*	VD01	S3	4	*Pichia manshurica* (S3)
									Saccharomyces cerevisiae (S3)
									Sugiyamaella novakii (G1-G5)
									Trigonopsis cantarellii (S3)
Af146 (23)	10.00	164.7	Mature	10 August, 2019	*Q. variabilis*	VD01	S3	≥5	*Candida* sp. 3

									Lachancea fermentati (S3)<br>*Scheffersomyces coipomensis*<br>*Scheffersomyces stipitis* (G1, G4)<br>*Sugiyamaella xiaguanensis*<br>Unidentified
Af106 (28)	9.06	130.1	Immature	21 June, 2019	*Q. variabilis*	VB01	Not on a sap patch	1	*Zygosaccharomyces* sp.
Af107 (5)	8.85	140.5	Mature	21 June, 2019	*Q. variabilis*	VB01	Non-recorded sap patch	0	n.a.
Af114 (18)	8.64	90.9	Immature	2 July, 2019	*Q. variabilis*	VA04	A270	1	*Cryptococcus podzolicus* (G1-G3)
Af120 (12)	9.49	119.4	Mature	10 July, 2019	*Q. variabilis*	VE04	A333	0	n.a.
Af117 (11)	9.06	123.7	Mature	10 July, 2019	*Ulmus davidiana* var. *japonica*	Not recorded	Non-recorded sap patch	0	n.a.

[a] Sampling date of sap for yeast isolation: 29 July, 2019 (S1), 2 August, 2019 (S2), and 10 August, 2019 (S3).

[b] Samples of larval gallery (G1-G5) and sap (S1-S3).

Supplementary Table S6. Yeasts isolated from the hindguts of *Aegus subnitidus* obtained from fermented sap patches of trees.

Individual ID (no. in Fig. 2c)	Yeast in hindguts	
	Number of species	Species (other isolation sources in environments)[a]
Af135 (29)	6	*Candida* sp. 5 (S1)
		Candida sp. 6 (S1)
		Ogataea sp. (S1)
		Pichia sp. 1 (S1)
		Pichia sp. 2 (S1)
		Prototheca sp. 4
Af140 (17)	≥8	*Lachancea fermentati* (S3)
		Pichia manshurica (S3)
		Prototheca sp. 1 (S2)
		Prototheca sp. 2 (S2)
		Prototheca sp. 3
		Sporopachydermia quercuum (S2)
		Sporopachydermia sp. (S2)
		Zygosaccharomyces sp.
		Unidentified
Af145 (25)	6	*Lachancea fermentati* (S3)
		Pichia manshurica (S3)
		Saccharomyces cerevisiae (S3)
		Saccharomycodes ludwigii (S3)
		Zygosaccharomyces pseudobailii
		Zygosaccharomyces sp.
Af146 (23)	≥10	*Lachancea fermentati* (S3)
		Pichia manshurica (S3)
		Priceomyces carsonii (S3)
		Saccharomyces cerevisiae (S3)
		Saccharomycodes ludwigii (S3)
		Schizosaccharomyces japonicus
		Starmerella sp.
		Taphrinomycotina sp.

	Trigonopsis cantarellii (S3)
	Zygosaccharomyces sp.
	Unidentified

[a] Samples of sap (S1-S3).
